# Extra-Cellular Matrix Proteins Induce Matrix Metalloproteinase-1 (MMP-1) Activity and Increase Airway Smooth Muscle Contraction in Asthma

**DOI:** 10.1371/journal.pone.0090565

**Published:** 2014-02-28

**Authors:** Natasha K. Rogers, Debbie Clements, Arundhati Dongre, Tim W. Harrison, Dominic Shaw, Simon R. Johnson

**Affiliations:** Division of Respiratory Medicine and Respiratory Research Unit, University of Nottingham, Nottingham, England, United Kingdom; National Center for Scientific Research Demokritos, Greece

## Abstract

Airway remodelling describes the histopathological changes leading to fixed airway obstruction in patients with asthma and includes extra-cellular matrix (ECM) deposition. Matrix metalloproteinase-1 (MMP-1) is present in remodelled airways but its relationship with ECM proteins and the resulting functional consequences are unknown. We used airway smooth muscle cells (ASM) and bronchial biopsies from control donors and patients with asthma to examine the regulation of MMP-1 by ECM in ASM cells and the effect of MMP-1 on ASM contraction. Collagen-I and tenascin-C induced MMP-1 protein expression, which for tenascin-C, was greater in asthma derived ASM cells. Tenascin-C induced MMP-1 expression was dependent on ERK1/2, JNK and p38 MAPK activation and attenuated by function blocking antibodies against the β1 and β3 integrin subunits. Tenascin-C and MMP-1 were not expressed in normal airways but co-localised in the ASM bundles and reticular basement membrane of patients with asthma. Further, ECM from asthma derived ASM cells stimulated MMP-1 expression to a greater degree than ECM from normal ASM. Bradykinin induced contraction of ASM cells seeded in 3D collagen gels was reduced by the MMP inhibitor ilomastat and by siRNA knockdown of MMP-1. In summary, the induction of MMP-1 in ASM cells by tenascin-C occurs in part via integrin mediated MAPK signalling. MMP-1 and tenascin-C are co-localised in the smooth muscle bundles of patients with asthma where this interaction may contribute to enhanced airway contraction. Our findings suggest that ECM changes in airway remodelling via MMP-1 could contribute to an environment promoting greater airway narrowing in response to broncho-constrictor stimuli and worsening asthma symptoms.

## Introduction

Asthma is a lung disease characterised by airway inflammation, bronchial hyperresponsiveness and variable airway obstruction. Chronic inflammation leads to a series of structural airway changes collectively termed airway remodelling, which lead to enhanced airway contraction and eventually fixed airflow obstruction. Changes observed in airway remodelling include epithelial desquamation, goblet cell hyperplasia, increased airway smooth muscle (ASM) mass, thickening of the reticular basement membrane and abnormal extracellular matrix (ECM) deposition. The ECM is abnormal in terms of composition and quantity, with increased expression of collagens, biglycan, elastin, fibronectin, hyaluronan, laminin-β2, lumican, tenascin-C and versican when compared with normal airways [1]–[5].

Matrix metalloproteinase-1 (MMP-1) is a collagenase, which is minimally expressed in normal lung tissue [6]–[9]. However, in patients with asthma, MMP-1 protein is present in the small airways and lung parenchyma. In BAL fluid, MMP-1 mRNA is directly correlated with airway obstruction. These observations suggest that collagenase expression is associated with airway narrowing and asthma symptoms although the mechanisms for this are unclear [7], [10], [11]. We and others have previously implicated ECM proteins as active mediators of airway remodelling with specific effects on airway epithelial integrity and repair, ASM growth, differentiation, survival, synthetic function, migration and phenotype [12]–[17]. As MMPs are regulated by ECM proteins in a number of systems, we hypothesised that the altered ECM in asthma may increase the expression and activity of MMPs and contribute to the asthma phenotype.

The relationship between ECM deposition, MMP-1 expression and airway function is not understood, although interestingly, collagenase treatment reduces passive tension and increases muscle shortening in human bronchial smooth muscle strips [18]. Collagenase treatment of *ex vivo* lung slices causes spontaneous airway narrowing [19] and inhalation of collagenase, increases bronchial hyperresponsiveness in rodent models of asthma [20], [21]. *In vitro* models of airway contraction also show that exogenous administration of MMP-1 can enhance airway contraction and that the pro-contractile effects of the Th2 cytokines IL-4 and IL-13 are MMP-1 dependent [22], [23]. Collectively these findings suggest that airway remodelling and ECM deposition could contribute to worsening airflow obstruction and bronchial hyperresponsiveness by mediating the aberrant expression of MMP-1 in the airways of patients.

Despite the potential importance of MMP-1 in asthma, few studies have examined its regulation in ASM cells. ASM derived MMP-1 mRNA and protein expression are upregulated by collagen-I [17], [22], platelet-derived growth factor [24], cyclic strain [25], leukotriene D4 [26] and combined treatment with TNF-α and IL-1β [27]. Understanding the roles of these asthma relevant regulators upon bioactive proteins including MMP­1, may provide novel therapeutic strategies to counter airway remodelling. We therefore examined the regulation of MMP-1 by the ECM proteins which are differentially expressed in remodelled airways and whether the resulting increase in MMP-1 activity could functionally contribute to the asthma phenotype.

## Materials and Methods

### Endobronchial Biopsies and Culture of ASM Cells

Endobronchial biopsies were obtained from patients with physician diagnosed asthma at British Thoracic Society stage II or III, without history of an exacerbation or change in therapy for at least 6 weeks [28]. Control endobronchial biopsy tissue was obtained from patients undergoing bronchoscopy for other reasons. Up to six endobronchial biopsies were taken from a first or second order sub-carina by fibre-optic bronchoscopy according to standard procedures. Biopsies were either formalin fixed and embedded in paraffin for histological assessment or used for culture of ASM cells as described previously [12]. ASM cells were maintained at 37°C in a humidified incubator in 95% air/5% CO_2_ and subcultured in DMEM (Sigma-Aldrich) supplemented with 10% FCS, penicillin (50 U/ml) and streptomycin (50 µg/ml). Cells were used between passage four and eight. A minimum of three asthma donors and three control donors were used for all experiments which were performed independently on at least three occasions. The use of both ASM cells and biopsy tissue was approved by the Nottingham Research Ethics Committee and written, informed consent was obtained from all patients.

### Coating Culture Plates with ECM Proteins

Tissue culture plates were incubated overnight with a 10 µg/ml solution of collagen-I (Sigma-Aldrich, #C7774), collagen-IV (Sigma-Aldrich, #C6746), fibronectin (Sigma-Aldrich, #F0895), laminin (Sigma-Aldrich, #L6274) or tenascin-C (#CC065, Millipore). Wells were washed with PBS, blocked with 1% BSA/PBS and again washed with PBS as previously described [14]. For cell derived ECM preparations, ASM cells from control and asthma derived cells were cultured in 12-well culture plates for 14 days, media aspirated and cells removed using ammonium hydroxide (50 mM) and Triton X-100 (0.05%). Following incubation with DNase I (20 U/ml) plates were washed with PBS and stored overnight (4°C). This preparation results in a cell free bioactive ECM coating [29]. Plates were allowed to warm to room temperature prior to seeding serum starved ASM cells, and cell culture supernatants were harvested after 24 hours.

### ECM Stimulation of ASM Cells

**Figure 1:**
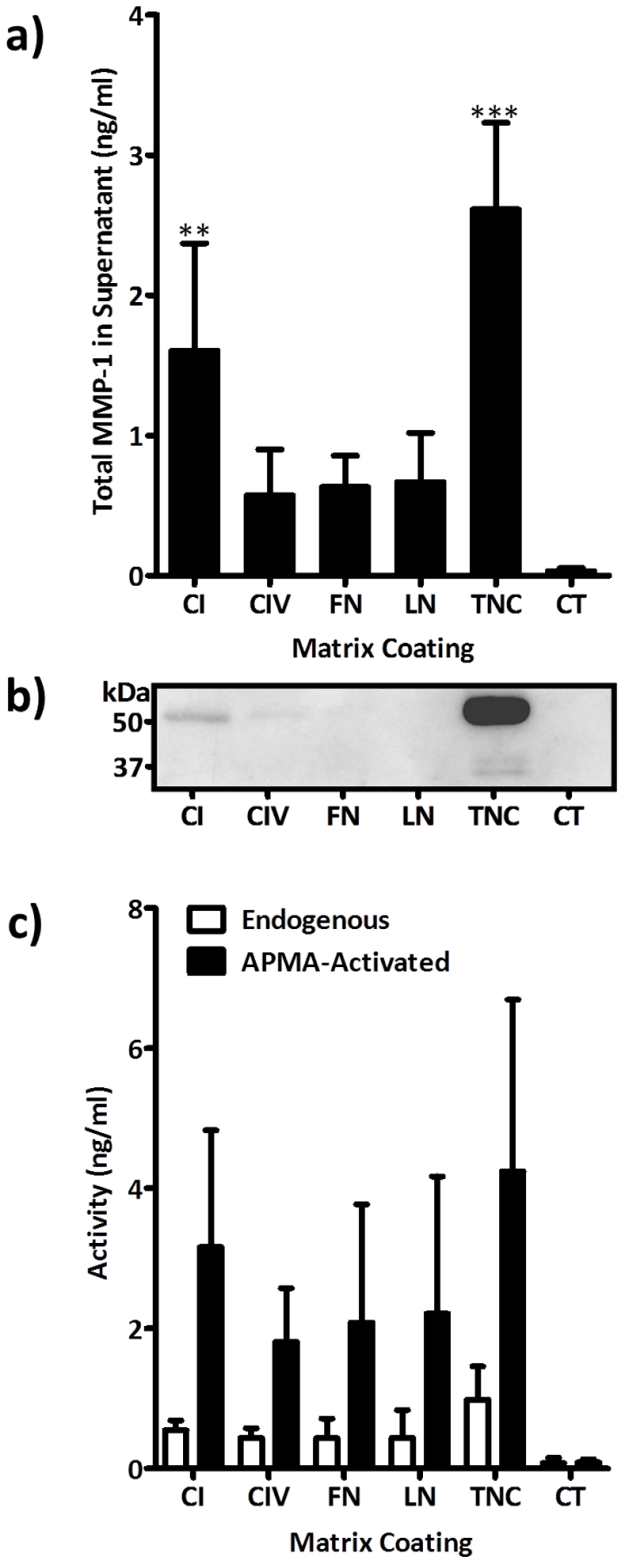
Induction of MMP-1 protein and activity by ECM proteins. Serum starved ASM cells from three separate donors were seeded on tissue culture plastic coated with ECM proteins (10 µg/ml) for 24 hours. (a) ASM derived supernatants were assayed for total MMP-1 expression by ELISA, (b) latent and active MMP-1 by western blotting and (c) active and total MMP-1 expression using an MMP-1 specific activity assay. Differences in MMP-1 protein expression were assessed for statistical significance by 1-way ANOVA with *post-hoc* Dunnett’s multiple comparison test (**P<0.01. ***P<0.001). Abbreviations: APMA, 4-aminophenylmercuric acetate; CI, collagen-I; CIV, collagen-IV; CT, control; FN, fibronectin; LN, Laminin; TNC, tenascin-C.

Experiments for determining MMP-1 secretion were initially performed by seeding serum-starved ASM cells in serum-free media onto tissue culture plates pre-coated with 10 µg/ml ECM proteins as previously described [30] (Figure 1). MMP-1 expression induced by this method was not significantly different (n = 3, p<0.05) from that induced by the addition of ECM to the culture medium of cells adherent to tissue culture plastic and all subsequent experiments were performed using soluble ECM proteins. Unless otherwise stated, ASM cell culture supernatant was harvested after 24 hours. An MTT assay was performed in conjunction with each experiment according to a previously described method [31] to ensure that ECM proteins did not affect cell number or have toxic effects. None of the ECM treatments altered cell viability or increased cell number (data not shown).

### MMP-1 Protein and Activity Determination

Total MMP­1 protein levels in cell culture supernatants, which includes pro, active and inhibitor bound MMP-1, were quantified by ELISA (Human Total MMP-1 DuoSet; DY901; R&D Systems) and MMP activity using an MMP-1 specific activity assay (Human Active MMP-1 Fluorokine E Kit; F1M00; R&D Systems) according to manufacturer’s instructions. For specific activity assay, samples were assayed with and without the addition of 4-aminophenylmercuric acetate (APMA) which activates pro-MMPs [32]. Total MMP-1 was inferred from the value for MMP-1 activity obtained after incubation of the samples with APMA. Pro- and active MMP-1 species were differentiated by casein zymography and immunoblotting in culture supernatants concentrated ten-fold using Vivaspin 500 columns with recombinant human pro-MMP­1 as a positive control.

For immunoblotting, equal quantities of supernatant were diluted with 5x reducing gel loading buffer (1 M Tris, 0.1% SDS (w/v), 0.08 DTT (w/v), 0.01% bromophenol (w/v) blue, 55% glycerol (w/v)) heated to 95°C and spun at 13,000rpm for 3 minutes. Samples were resolved by electrophoresis on 10% SDS-polyacrylamide gels and blotted onto a PVDF membrane. Membranes were probed with a polyclonal anti-MMP-1 antibody (Merck, #444209, 1/500) and a secondary HRP-conjugated polyclonal goat anti-rabbit IgG antibody. Signals were visualized using the ECL Western Blotting Detection Kit (Amersham Biosciences). For casein zymography, supernatants were diluted with an equal volume of 2x Novex sample buffer (Invitrogen) and resolved by electrophoresis on 12% casein gels (Invitrogen) using the Novex buffer system. Gels were incubated in Novex renaturing buffer after electrophoresis followed by an overnight incubation in Novex developing buffer at 37°C. Gels were incubated in colloidal blue stain solution and destained overnight in water. Images were acquired using SynGene GeneSnap v7.04 g attached to a GeneGenius image acquisition system (Synoptics, Cambridge, UK) [33].

### Quantification of Gene Expression

RNA was extracted from ASM cells using an RNeasy Mini Kit (Qiagen) and contaminating genomic DNA removed using DNAse I (Qiagen). RNA was reverse-transcribed into cDNA using the SuperScript II First-Strand Synthesis System for RT-PCR (Invitrogen) with random hexamer primers according to manufacturer’s instructions. To determine the relative gene expression levels of *MMP-1*, *COL1A1* and *TNC*, cDNA was amplified by quantitative real-time PCR using the Brilliant II SYBR Green QPCR master mix (Agilent Technologies) with the primers listed below. Reactions were performed in triplicate and the specificity of the primers was confirmed for every PCR run by dissociation curve analysis. Expression levels of target genes were determined relative to a housekeeping gene GAPDH using the comparative CT (2^−ΔΔCT^) method. The primers utilised are as follows:

*COL1A1*: F5′­GAACGCGTGTCATCCCTTGT­3′ R5′­GAACGAGGTAGTCTTTCAGCAACA­3′ *GAPDH*: F5′­GGTCTCCTCTGACTTCAACA­3′ R5′­AGCCAAATTCGTTGTCATAC­3′ *MMP1:*F5′­GAGCAAACACACTGACCTACAGGA­3′ R5′­TTGTCCCGATGATCTCCCCTGACA­3′ *TNC:* F5′­CCACAATGGCAGATCCTTCT3­3′ R5′­GTTAACGCCCTGACTGTGGT­3′.

### Immunodetection of Protein Phosphorylation

Protein phosphorylation was performed as previously described [34]. ASM cells were lysed by 30 minute incubation in ice-cold PhosphoSafe Extraction Reagent (Merck) containing a Complete Protease Inhibitor Cocktail (Roche). Lysates were harvested and insoluble cell debris was removed by centrifugation at 13,000×*g* for 10 minutes at 4°C. Total cell lysate protein content from the soluble fractions was quantified by the Bradford based Bio-Rad protein assay kit to normalize protein content. Samples were diluted in 5x reducing gel loading buffer (1 M Tris, 0.1% SDS (w/v), 0.08 DTT (w/v), 0.01% bromophenol blue (w/v), 55% glycerol (w/v)), heated to 95°C and spun at 13,000rpm for three minutes. Samples were resolved by electrophoresis on 10% SDS-polyacrylamide gels and blotted onto a PVDF membrane. Membranes were initially probed for: phospho-Akt (Ser473) (#4060, 1/1000), phospho-Erk1/2 (Thr202/Tyr204) (#9106, 1/1000), phospho-p38 MAPK (Thr180/Tyr182) and Phospho-SAPK/JNK (Thr183/Tyr185) (#9251, 1/200). Following incubation with primary antibodies, blots were incubated with a secondary HRP-conjugated polyclonal antibody. Signals were visualized using the ECL Western Blotting Detection Kit (Amersham Biosciences). Subsequently membranes were stripped using Restore Western Blot Stripping Buffer (Pierce) and reprobed for total expression of: Akt (#2920, 1/1000); p44/42 MAPK (Erk1/2) (#9102, 1/1000); p38 MAPK (#9212, 1/500); phospho-p38 MAPK (Thr180/Tyr182) (#4511, 1/500) and SAPK/JNK (#9258, 1/200) which were used for normalization. All antibodies were purchased from cell signaling technologies. Alternatively, after 20 min of stimulation with soluble tenascin-C (10 µg) ASM cell lysates (200 µg) were analyzed by immunoblot for a panel of multiple phosphorylated proteins (Human Phospho-MAPK Array Kit, #ARY002B, R&D), according to the manufacturer’s instructions.

### Inhibition of Protein Phosphorylation

ASM cells were treated with phosphorylation inhibitors (SP600125, 10 µmol; U0126, 10 µmol; NS23766, 100 µmol; SB208350, 10 µmol; rapamycin 10 nmol; wortmanin 100 nmol; SB216763 10 µmol) for 30 minutes prior to stimulation with tenascin-C (10 µg/ml). Cell culture supernatant was collected after 24 hours and assayed by ELISA for total MMP-1 concentration. All inhibitors were purchased from Merck, apart from SB216763, which was sourced from Sigma.

### Receptor Expression and Inhibition

Cell surface expression of receptors was determined by flow cytometry using methods previously described [35]. Briefly, ASM cells were cultured in T75 cm^2^ tissue culture flasks, detached with Accutase® Cell Dissociation Reagent (Sigma), pelleted and re-suspended in ice cold 2% BSA/PBS. Cells were incubated on ice for 45 minutes with antibodies against integrin subunits β1 (R&D, MAB17781) and β3 (Millipore, MAB2023Z), the αvβ5 integrin (VWR, AB24694) and toll like receptor 4 (TLR4) (R&D, AF1478). Polyclonal goat IgG (AB108C, R&D) was used as an isotype control for TLR4 experiments and a monoclonal mouse IgG1 (MABC002, Millipore) as isotype control for integrin experiments. All antibodies were used at 10 µg/ml. Cells were washed twice in ice-cold 2% BSA/PBS solutions and incubated with the appropriate Alexa-Fluor 488 conjugated secondary antibody (Invitrogen). Cells were washed again, and fixed in a 1% formaldehyde/PBS solution, which was removed prior to analysis. Flow cytometry was performed using a Coulter FC 500 Flow Cytometer (Beckman Coulter) and data analysed using flow cytometry data analysis software (Weasel, The Walter and Eliza Hall Institute of Medical Research). Integrin antibodies were also used for receptor blocking experiments. Experiments were performed on serum-starved ASM cells seeded on uncoated tissue culture plastic. ASM cells were treated with antibodies against the β1, β3 and the αvβ5 integrin (5, 10 or 20 µg/ml) or monoclonal mouse IgG1 isotype control for 1 hour prior to stimulation with tenascin-C (10 µg/ml). Cell culture supernatant was collected after 24 hours and assayed by ELISA for total MMP-1 concentration. For each concentration of each antibody type, MMP-1 concentration is expressed as a percentage of that elicited by cells stimulated with the isotype control at the corresponding concentration. Expression levels were normalised to isotype control (at equivalent concentration, stimulated with tenascin-C (10 µg/ml)).

### Localisation of MMP-1 and Tenascin-C in Endobronchial Biopsies

Immunohistochemistry was performed on sections cut from formalin-fixed tissue embedded in paraffin blocks. Slides were dewaxed in Histoclear (Fisher-Scientific) and rehydrated in a series of graded alcohols. Slides were washed in water and PBS and immersed in a 3% hydrogen peroxide solution for 10 minutes. To block nonspecific binding sites, slides were incubated in horse serum (2.5%) for 30 minutes. Sections were treated with anti-MMP-1 (Merck, #444209, 1∶200), anti­tenascin­C (Biohit, #610003, 1∶200) or isotype control antibodies overnight at 4°C in an airtight humidified chamber. After washes with water and PBS, primary antibodies were detected with ImmPRESS Universal Antibody (anti-mouse Ig/anti-rabbit Ig, peroxidase; Vector Laboratories, #MP-7500) and visualized using the Vector VIP substrate kit (Vector Laboratories, #SK-4600) and counterstained with haematoxylin [35].

### *In vitro* Contraction Assays

Serum starved ASM cells were suspended in a neutralized solution of bovine collagen-I (Sigma-Aldrich; C4243) and 10x DMEM (Sigma-Aldrich) and cast in 24-well tissue culture plates to create 7 mm-thick three dimensional (3D) collagen gels. Gels were allowed to set for 24 hours before being overlaid with DMEM. After 16 hours, gels released from wells and transferred to 6-well plates containing DMEM/F12 (Sigma-Aldrich) [36]. Contraction was stimulated with bradykinin (Sigma-Aldrich). Gel contraction was quantitated over 60 minutes using Genesnap camera (Genetools, Syngene, and Cambridgeshire, UK) with the reduction in initial gel area quantitated using Image J (http://rsb.info.nih.gov/ij). In experiments involving MMP inhibition, gels were treated with 10 µM ilomastat (Sigma-Aldrich) 1 hour prior to bradykinin stimulation.

### siRNA Inhibition of MMP-1

siRNA was used as described with the following modifications [12]. ASM cells were transfected with Lipofectamine 2000 (Invitrogen), Opti-MEM and 20 nM of siRNA (Selleck Chemicals) according to manufactures instructions. Two separate MMP-1 specific siRNA sequences were used, both of which caused a similar reduction in MMP-1 protein assessed by ELISA. An unrelated control siRNA sequence transfected using the same protocol had no effect on MMP-1 expression. For all siRNA experiments, MMP-1 specific sequences were compared with the control siRNA. Knockdown of MMP-1 expression was confirmed at 24 hours and at the end of contraction experiments by MMP-1 ELISA.

### Statistical Analysis

Statistical analysis was performed using the Prism 5 for windows (version 5.02, 1992-2009 GraphPad Software, Inc. US). Data are presented as mean ± SEM. A p-value <0.05 was considered to be significant.

## Results

### MMP-1 is Induced by Extracellular Matrix Proteins

To determine if MMP-1 could be regulated by ECM proteins of relevance to airway remodelling: ASM cells were cultured on collagen-I, collagen-IV, fibronectin, laminin and tenascin-C. Although all matrix substrates enhanced total MMP-1 protein in culture supernatants, there were robust and significant rises in MMP-1 in ASM grown upon collagen­I and tenascin­C (figure 1a). This finding was supported by western blot analysis of culture supernatants showing increased expression of the 54 kDa pro-form of MMP-1 particularly in collagen-I and tenascin-C treated cells. In tenascin­C treated cells the two active species of MMP-1 were also detected (figure 1b). We then used a specific MMP-1 activity assay which showed increased active and total MMP-1 in a similar pattern to the ELISA, particularly in tenascin-C treated cells (figure 1c). Comparable trends were observed for all donors studied.

### Tenascin-C dose Dependently Enhances MMP-1 Expression and Activity

**Figure 2:**
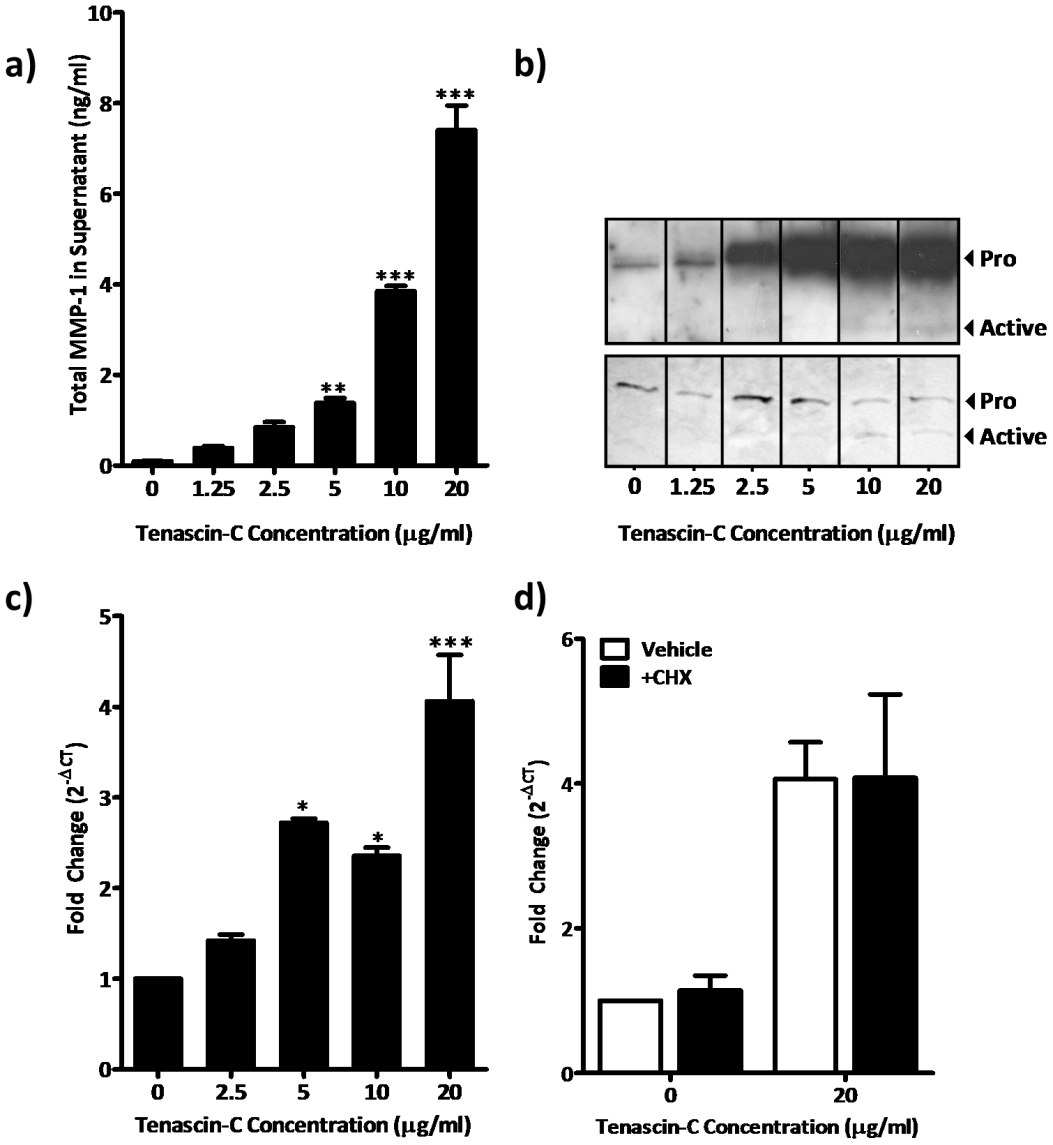
Induction of MMP-1 protein and activity by tenascin-C is concentration dependent and transcriptionally mediated. ASM cells from three different donors were stimulated with various concentrations of soluble tenascin-C for 24 hours. (a) ASM cell supernatant was assayed for total MMP-1 expression by ELISA; (b) latent and active MMP-1 by western blotting (top panel) and casein zymography (lower panel, the latter image is inverted to aid visualisation). Differences in MMP-1 protein expression were assessed for statistical significance by 1-way ANOVA with *post-hoc* Dunnett’s multiple comparison test (**P<0.01. ***P<0.001). (c) MMP-1 mRNA from three donors stimulated with various concentrations of soluble tenascin-C levels are expressed as fold change in cycle threshold number relative to untreated cells (normalised to GAPDH) and compared by 1-way ANOVA with *post-hoc* Dunnett’s Multiple comparison test. (d) ASM cells treated with 20 µg/ml tenascin-C or vehicle were cultured with and without cycloheximide and MMP-1 mRNA analysed by paired one-tailed t-test (*P<0.05. ***P<0.001). Abbreviations: CHX, cycloheximide; ct, cycle threshold.

As tenascin-C strongly induced MMP-1 expression and activity and is expressed in the airways of patients with asthma, tenascin-C was studied in detail. There was a concentration dependent increase in MMP-1 protein in supernatants of ASM cells cultured with tenascin-C as determined both by ELISA and western blotting (figure 2a, b). Active 45 kDa MMP-1 became detectable using western blotting when cells were exposed to concentrations of tenascin-C of 10 µg/ml or greater (figure 2b). Casein zymography, a substrate based assay, was used to further investigate these findings, caseinolytic activity was observed corresponding to the molecular mass of pro and active MMP-1 in supernatants from tenascin-C stimulated cells (figure 2b). We next used real time PCR to determine if tenascin-C also elicited a change in levels of MMP-1 mRNA. Tenascin-C dose dependently increased the expression of *MMP1* mRNA at 6 hours (figure 2c). Addition of cycloheximide to ASM cultures had no effect on mRNA induction by tenascin-C suggesting this was a direct effect on the *MMP1* promoter which did not involve synthesis of other proteins (figure 2d).

### Tenascin-C Mediated Induction of MMP-1 is via JNK, p38 and ERK1/2 MAPKs

**Figure 3:**
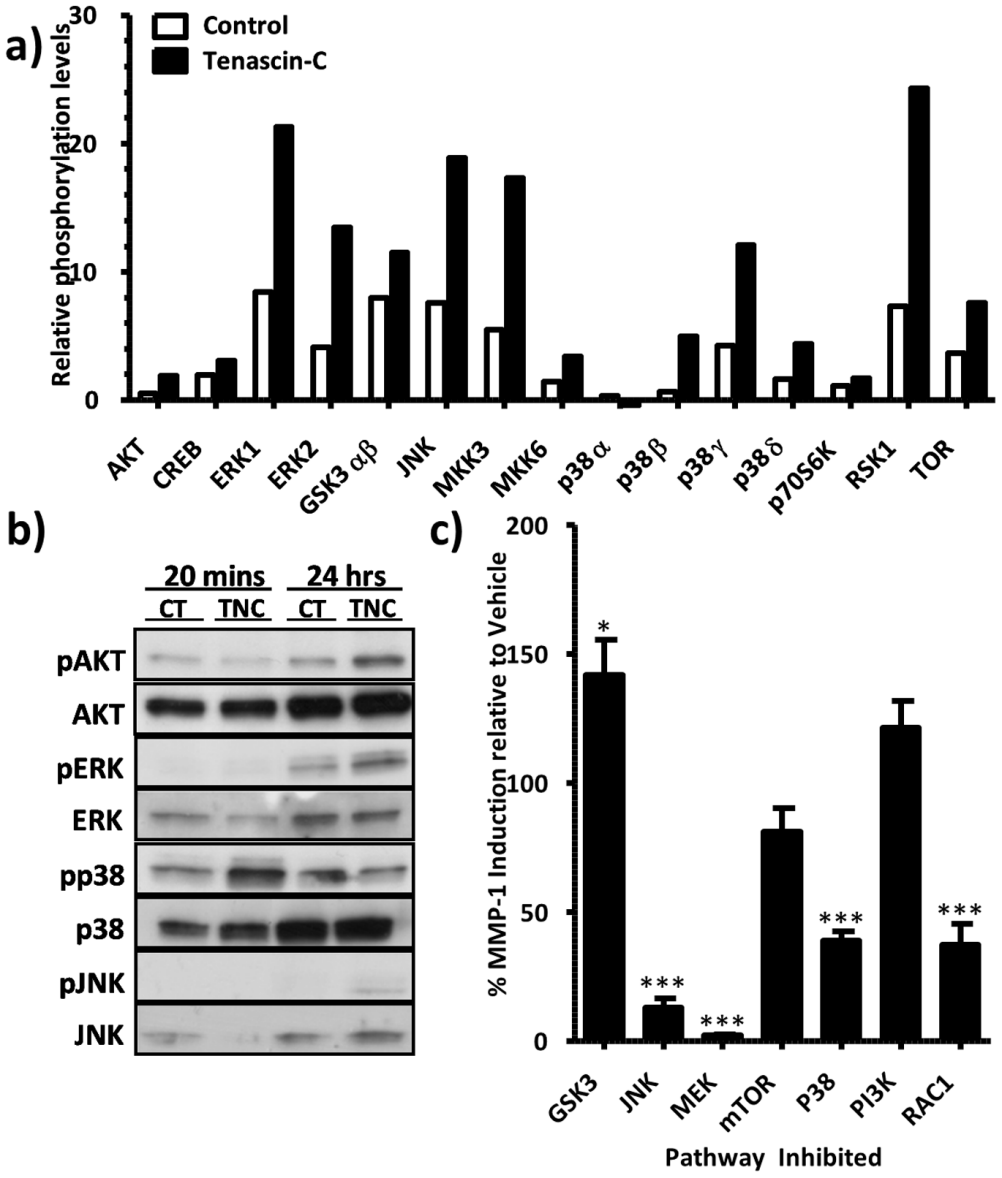
Induction of MMP-1 protein by tenascin-C is ERK1/2, JNK and p38 dependent. (a) ASM cells were stimulated with soluble tenascin-C (10 µg/ml). Lysates were harvested at 20 minutes and assayed by immunoblot for a panel of phosphorylated signalling proteins using proteome profiler. (b) ASM cells from three normal donors were stimulated with soluble tenascin-C (10 µg/ml). Lysates were harvested at 20 minutes and 24 hours, with levels of total and phosphorylated signalling proteins assayed by Western Blotting. (c) ASM cells from three normal donors were incubated for 30 minutes with inhibitors of JNK (SP600125, 10 µmol), MEK1/2 (U0126, 10 µmol), Rac1 (NS23766, 100 µmol), p38 (SB208350, 10 µmol), mammalian target of rapamycin complex 1 (rapamycin 10 nmol), PI3K (wortmanin 100 nmol) or glycogen synthase kinase αβ (GSK3αβ, SB216763 10 µmol) prior to stimulation with tenascin-C (10 µg/ml). Supernatant was harvested after 24 hours and assayed for MMP-1 protein. Expression levels were normalised to vehicle control (0.02% DMSO, tenascin-C (10 µg/ml)) and analysed using a 1-way ANOVA with *post-hoc* Dunnett’s multiple comparison test (*P≤0.05. ***P<0.001).

**Figure 4:**
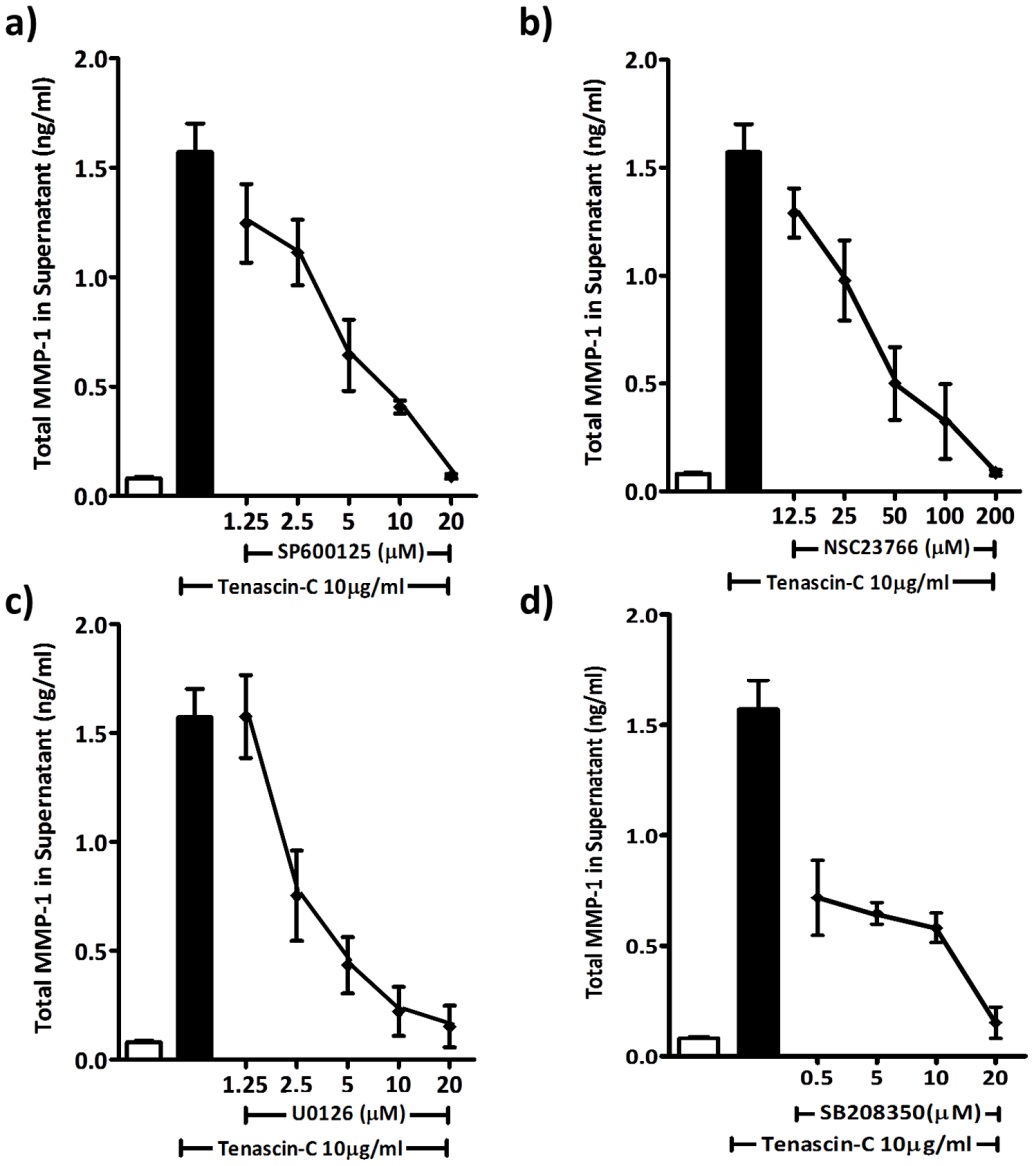
Selective inhibitors of Rac1, JNK, ERK1/2 and p38 dose dependently block the induction of MMP-1 by tenascin-C. ASM cells from three normal donors were incubated with (a) U0126; (b) SB203580; (c) NSC23766; (d) SP200125 at indicated concentrations, prior to stimulation with soluble tenascin-C (10 µg/ml). Supernatant was harvested after 24 hours and assayed for MMP-1 protein. The white bar represents the vehicle control (0.02% DMSO) and the black bar represents the stimulated vehicle control (0.02% DMSO and tenascin-C (10 µg/ml)).

To determine the signalling pathways regulating tenascin-C induced MMP-1 secretion, we took a dual approach examining the effect of a range of signalling inhibitors on tenascin-C induced MMP-1 protein expression and the intermediates phosphorylated by tenascin-C after at 20 minutes and 24 hours. p38 MAPK was phosphorylated after 20 minutes of tenascin-C exposure whereas Akt, MEK 1/2 and JNK were phosphorylated after 24 hours of tenascin-C exposure as shown by phosphoarray and western blotting (figure 3a & b). Enhanced expression of MMP-1 was blocked by inhibitors of JNK (SP600125, 10 µmol), MEK1/2 (U0126, 10 µmol), Rac1 (NS23766, 100 µmol) and p38 (SB208350, 10 µmol) (figure 3c). Inhibition of mammalian target of rapamycin complex 1 (rapamycin 10 nmol), PI3K (wortmanin 100 nmol) and glycogen synthase kinase-3 αβ (SB216763 10 µmol) did not attenuate tenascin-C induced MMP-1 expression (figure 3c). We then went on to show that U0126, SB208350, NS23766 and SP600125 had dose dependent effects of on the inhibition of tenascin-C induced MMP-1 production (figure 4).

### Tenascin-C Induces MMP-1 Expression via β1 and β3 Integrins

**Figure 5:**
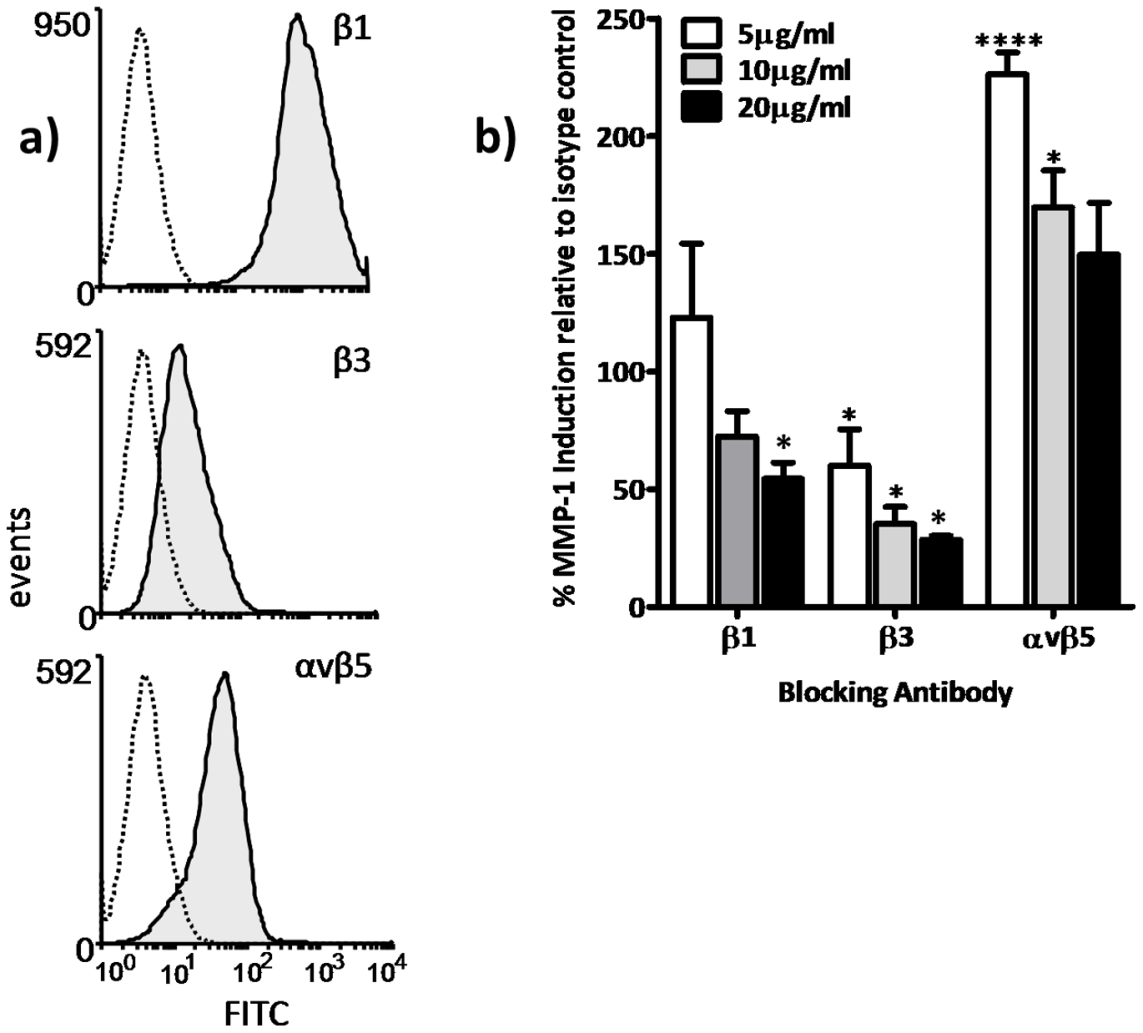
Tenascin-C induced MMP-1 secretion is mediated by the β1 and β3 integrin subunits. (a) ASM cells processed for flow cytometry were incubated with antibodies against the integrin subunits β1, β3 and the αvβ5 receptor prior to incubation with a fluorescently conjugated secondary antibody. Histogram depicts fluorescence intensity detected by flow cytometry (one representative experiment of three shown) for antigens (shaded) and their respective isotype control (broken line). (b) ASM cells were incubated with the same antibodies as listed above prior to stimulation with tenascin-C (10 µg/ml). Supernatant was harvested after 24 hours and assayed for MMP-1 protein. Expression levels analysed using a 2-way ANOVA with *post-hoc* Dunnett’s Multiple comparison test (*P≤0.05. ****P≤0.0001). Graph depicts results normalised to the isotype control (stimulated with tenascin-C (10 µg/ml)) at equivalent concentration, representative experiment of three separate donors is shown.

To determine which receptors were responsible for tenascin-C stimulated MMP-1 expression we performed flow cytometry for the candidate tenascin-C receptors expressed by ASM cells, TLR4 and β1, β3 and αvβ5 integrins. TLR4 was not expressed by unstimulated ASM (data not shown) but β1, β3 and αvβ5 were present on the majority of ASM cells (figure 5a). We next treated ASM with blocking antibodies against these integrin subunits and showed a dose dependent inhibition of tenascin-C stimulated MMP-1 expression for β1 and β3 but not αvβ5 when compared with an isotype control antibody (figure 5b).

### Tenascin-C Stimulated MMP-1 Expression is Enhanced in Asthma

**Figure 6:**
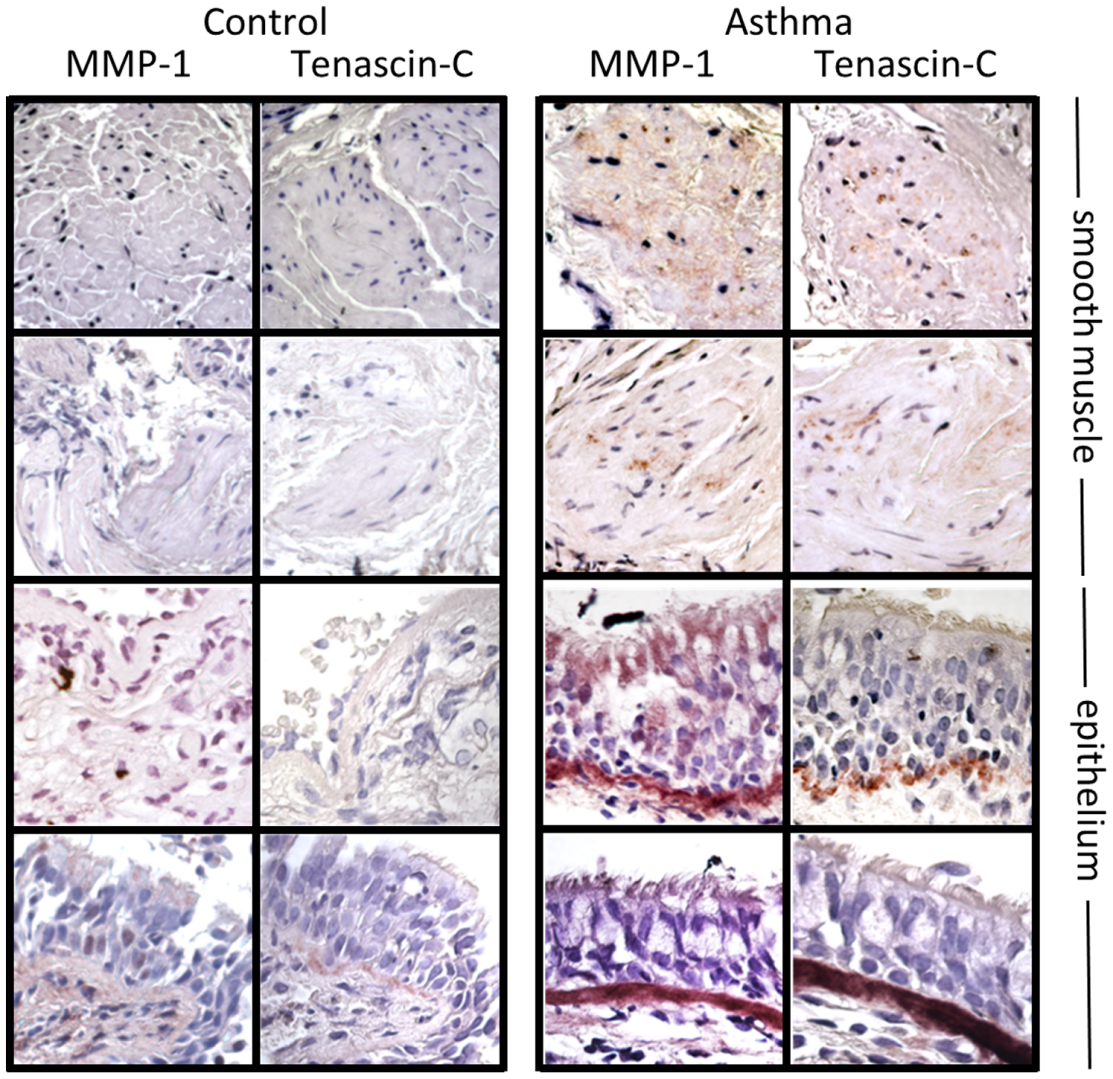
Expression of tenascin-C and MMP-1 in the airway. Endobronchial biopsies from seven patients with asthma and five controls stained for MMP-1 and tenascin-C. Representative images of smooth muscle bundles and epithelium are shown with positive staining visible as violet colouration. Original magnification ×100.

**Figure 7:**
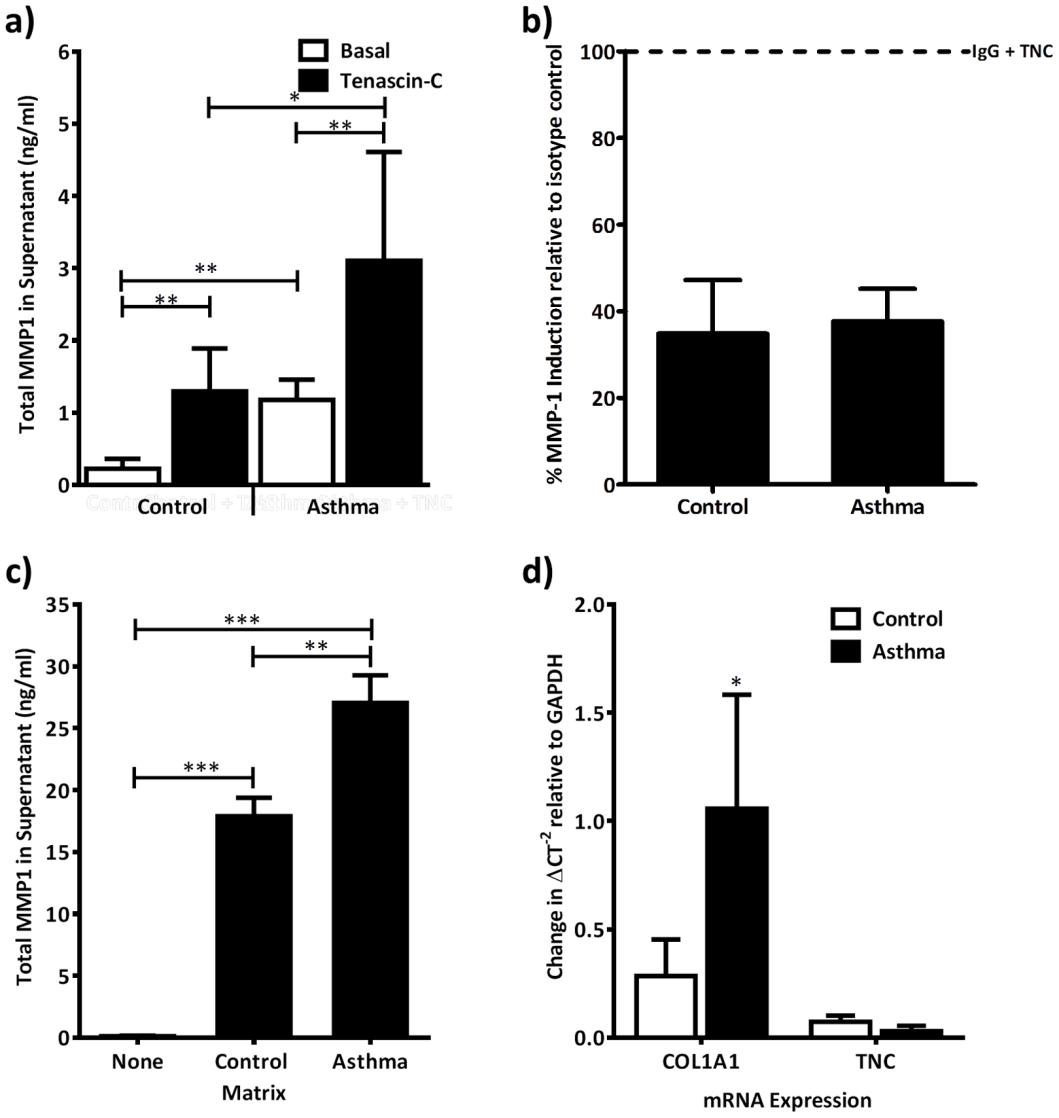
Asthma ASM cells secrete greater quantities of MMP-1 protein than control cells and control by ECM. (a) Six asthma derived and six control ASM cultures were incubated for 24 hours with and without the addition of soluble tenascin-C (10 µg/ml). The supernatant was harvested and levels of total MMP-1 assessed by ELISA. Expression levels were analysed using an unpaired one-tailed t-test (*P<0.05. **P<0.01). (b) ASM cells from four asthma and three control donors were incubated with a β3 integrin subunit function blocking antibody prior to stimulation with tenascin-C (10 µg/ml). Supernatant was harvested after 24 hours and assayed for MMP-1 expression. Expression levels for control and asthma ASM were separately normalised to an equivalent concentration of an isotype control, stimulated with tenascin-C (10 µg/ml). (c) Three control ASM cell cultures were seeded on ECM deposited by ASM cells from six asthma and six control donors. After 24 hours total MMP-1 was assessed by ELISA. Expression was analysed using a 1 way ANOVA with *post-hoc* Dunnett’s Multiple comparison test (**P<0.01. ***P≤0.001). (c) Serum starved ASM cells from four asthma and three control donors were seeded onto tissue culture plastic for six hours and TNC or COL1A1 mRNA assessed by qRT-PCR. Levels are expressed as mean fold change in cycle threshold number relative to GAPDH and compared by paired t-test (*P<0.05).

To determine if tenascin-C regulates MMP-1 in disease, we examined the expression of tenascin-C and MMP-1 in the airways from five control donors and seven patients with asthma. Neither tenascin-C nor MMP-1 protein was expressed in normal airways from non-smokers. In patients with asthma there was strong expression of both tenascin-C and MMP-1 in the sub-epithelial basement membrane and smooth muscle bundles (figure 6). Isotype control antibodies for both MMP-1 and tenascin-C caused no colour reaction (data not shown). We then compared MMP-1 expression in ASM cells obtained from six patients with asthma and six controls. Asthma derived ASM had higher basal expression of MMP-1 and higher tenascin-C stimulated MMP-1 levels than control ASM cells (figure 7a). Comparison of ASM cells from three control and four asthma donors showed no significant difference in expression of the β1, β3 and αvβ5 integrins. Further, the use of integrin blocking antibodies showed that inhibition of the β3 integrin subunit blocked MMP-1 production to the same degree as control cells (figure 7b).

As increased ECM deposition is a feature of airway remodelling in asthma, we hypothesised that asthma derived ASM may secrete increased levels of ECM proteins resulting in enhanced MMP-1 production by an autocrine mechanism. We therefore compared gene expression of tenascin-C and collagen-I in four asthma and three control ASM cell cultures by real-time PCR. Compared with control ASM, asthma derived cells had significantly increased collagen-I but similar levels of tenascin-C gene expression (figure 7c). To determine if this change in ECM expression by asthma derived ASM could be responsible for the enhanced MMP-1 secretion we generated ECM preparations from three control and five asthma derived ASM cultures. Both ECM preparations strongly induced MMP-1 expression in three unstimulated control ASM cultures when compared to tissue culture plastic. This induction was significantly greater on ECM derived asthma cells when compared with control ASM (figure 7d).

### MMP-1 Facilitates Airway Smooth Muscle Cell Contraction *in vitro*

**Figure 8:**
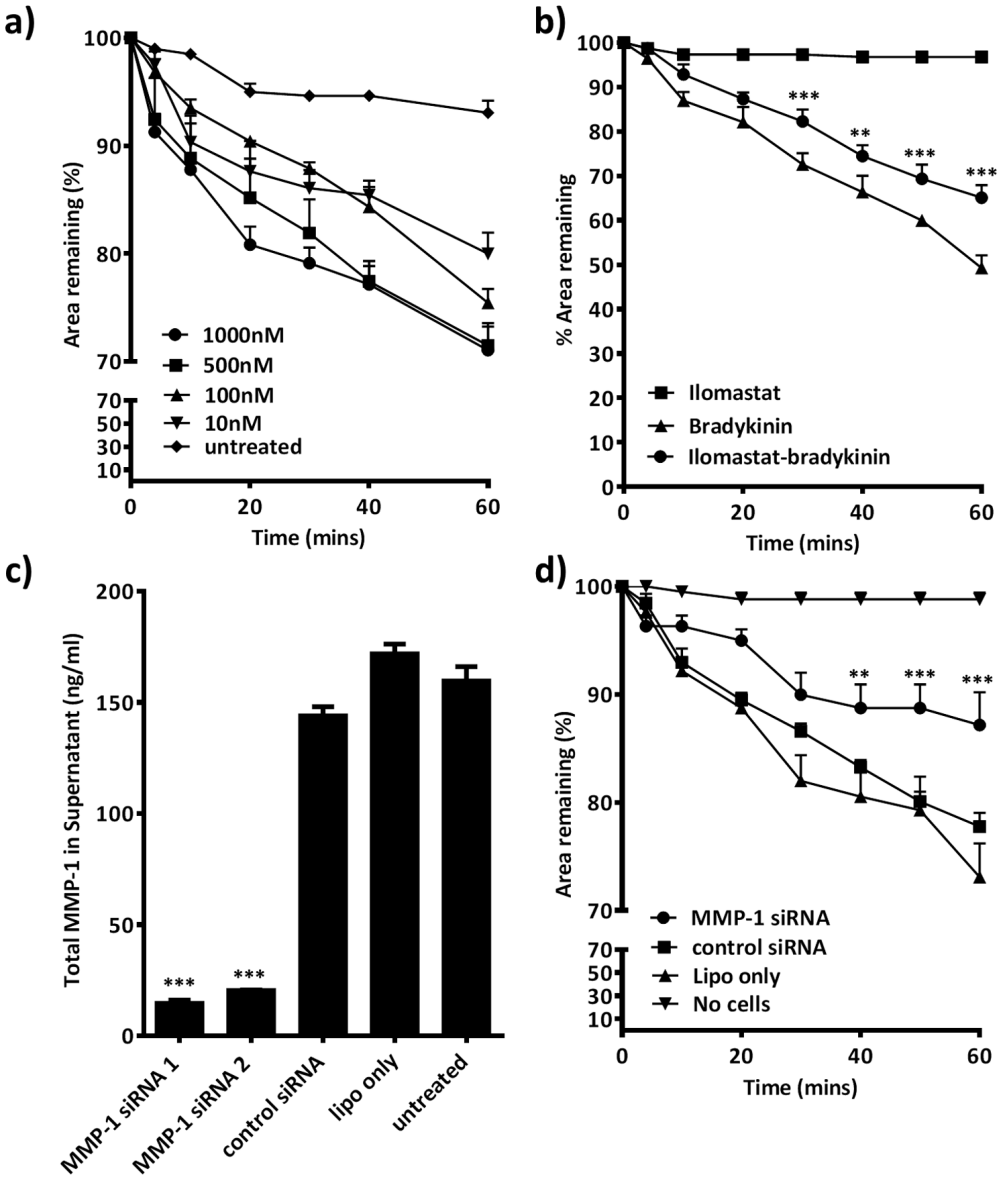
MMP expression and activation enhances ASM contraction. (a) Bradykinin-stimulated contraction of 3D collagen gels containing ASM cells is concentration dependent. (b) Ilomastat pretreatment (10 µmol) reduces bradykinin induced contraction when compared with vehicle control. Changes are analysed using a two way Repeated-Measures ANOVA with post-hoc bonferroni multiple comparison (c) siRNA against MMP-1 reduces MMP-1 expression in 3D collagen gel supernatants and analysed using a one way ANOVA with post-hoc Dunnet’s multiple comparison test (d) siRNA against MMP-1 reduces ASM contraction compared with transfection of a control siRNA and analysed by two way Repeated-Measures ANOVA with post-hoc bonferroni multiple comparison test. Graphs represent mean +/−SE of four replicate gels from three donors repeated on three occasions. (**P<0.01. ***P≤0.001).

Having shown that ECM proteins can induce the expression of MMP-1 by ASM, and that these proteins are co­localised in the airways of patients with asthma, we examined whether MMP-1 had functional consequences for airway contraction. We used an assay which examines contraction of ASM in 3D collagen gels. In initial experiments 3D ASM gels contracted in response to the ASM contractile agonists, serotonin, carbachol and bradykinin to a mean (SD) value of 82.7% (1.4), 87.3% (0.8) and 48.4% (8.8) of the original area after 60 minutes respectively (all p<0.005). There was a robust dose dependent contraction in response to bradykinin (figure 8a) which was used for further experiments. ASM cells suspended in 3D collagen gels strongly expressed MMP-1. Pre-treatment of gels with ilomastat (10 µmol) reduced bradykinin induced contraction when compared with vehicle controls (figure 8b). We then used siRNAs against MMP-1, which reduced MMP-1 expression in gel supernatants by eight fold compared with control siRNA (figure 8c). Transfection of ASM cells with this siRNA resulted in a significant reduction in gel contraction compared with control siRNA (figure 8d).

## Discussion

The data presented here demonstrate that the production and activation of MMP-1 by ASM is regulated by ECM proteins present in the airways of patients with asthma. Both collagen-I and tenascin-C enhanced MMP-1 expression with tenascin-C also inducing the active form of MMP-1. As the regulation of MMP-1 by collagens has been studied in detail previously [17], [37], [38] we focused on the effects of tenascin-C, which we have shown increased levels of latent and active MMP-1 protein via β3-integrin mediated MAPK signalling. Asthma derived ASM *in* *vitro* had higher basal expression levels of MMP-1 and upregulated MMP-1 expression in response to tenascin-C via a β3-integrin sensitive mechanism in common with normal cells. Asthma derived ASM cells produced more collagen-I under basal conditions than normal cells and ECM deposited by asthma derived cells had a greater capacity to induce MMP-1 secretion than control ECM. Of functional relevance to asthma, MMP-1, collagen-I and tenascin-C are co-located in the airways of patients with asthma and ASM derived MMP-1 facilitated the agonist induced ASM contraction *in vitro*. Our findings would suggest that increased MMP-1 in asthma biopsies and asthma ASM cells is the result at least in part of abnormal deposition of ECM. Previous work has suggested that pro-inflammatory cytokines including IL1β and TNFα [39], [40] and cyclic strain [25] also induce MMP-1 expression in ASM and are likely to be of relevance to asthma.

This is the first study to show tenascin-C can induce MMP-1 expression in primary human cells [41]–[43], and whilst αvβ3 integrin mediated MMP-1 induction has been documented [44], [45], our study demonstrates this response can also be initiated by tenascin-C. This mechanism involved tenascin-C dependent p38 phosphorylation; which had previously only been indirectly inferred by inhibitor studies [46]–[48]. Tenascin-C contains multiple cell interaction domains having differing effects in differing cell types [49]. Integrins α2β1 and αvβ3 are involved in many of these processes with the αvβ3 integrin interaction mediated by the peptide sequence SRRGDMS in endothelial cells implicating an RGD dependant mechanism [50]. In addition, the αvβ3 specifically binds tenascin-C via its fibrinogen-like domain [51].

Despite the presence of ECM proteins, particularly collagen-I and laminin within the normal airway, MMP-1 expression is minimal. In patients with both mild and fatal asthma there is both an increase in constitutively expressed ECM proteins and re-expression of proteins normally restricted to the developing lung including tenascin-C and some laminin isoforms. In these patients, we and others observed expression of MMP-1 within the ASM bundles [7]. As MMP-1 expression by ASM is regulated by ECM proteins in vitro, this indicates the complex ECM environment in the asthmatic airway has the potential to alter MMP-1 expression by the presence of tenascin-C and collagen-I. This is consistent with our findings that asthma derived ASM cells produce more MMP-1 and a functionally different ECM. Although we show that unstimulated asthma derived cells have similar tenascin-C gene expression to normal ASM, the Th_2_ cytokine IL-13, can strongly induce tenascin-C expression by ASM suggesting the asthma phenotype may induce ASM to synthesise tenascin-C [52], [53]. These observations suggest that in the remodelled airways of patients with asthma, ECM proteins act together to induce MMP-1 expression.

It is unknown whether MMP-1 expression confers pathogenic or protective effects in airway remodelling. A simple interpretation of its role is to function as a negative regulator of excessive ECM deposition. However, previous studies demonstrate potentially pro-remodelling roles for MMP-1, including promotion of ASM proliferation [7], [26]. Additionally, MMP processed collagen-I generates β3-integrin ligands which can induce tenascin-C expression in vascular smooth muscle [54], raising the possibility that tenascin-C induced MMP-1 induction may be a self-sustaining process. Ovalbumin challenge of sensitised tenascin-C knockout mice results in lower levels of airway reactivity, monocyte chemoattractant protein-1, IL-5, IL-13 and IgE in bronchoalveolar lavage fluid [55]. As specific features of airway remodelling have been associated with asthma severity [56], it suggests elements of airway remodelling may sustain airway inflammation and bronchial hyperresponsiveness. In our study ASM were derived from patients with asthma using inhaled corticosteroids. To our knowledge there are no data linking the use of these drugs with MMP-1 expression in asthma: it will be important to determine how corticosteroids affect MMP-1 expression both via direct effects and indirectly through the modulation of inflammatory mediators.

We sought to investigate the contractile function of ASM derived MMP-1. Contraction was assessed using a collagen gel contraction assay, which involves suspending ASM cells within a collagen-I matrix. We chose to use a ‘restrained’ matrix contraction model, which involves a period of culture with the gel attached to the culture surfaces, creating tension within the matrix. When the gel is released, further contraction occurs as mechanical stress dissipates [57]. Cells within the restrained 3D matrix develop pseudopodia and integrin-dependent collagen adhesions. Integrin engagement triggers structural and signalling protein translocation to the cell membrane and focal adhesion assembly. Gel contraction is dependent on actin polymerisation and the resulting force transferred through focal adhesions to the surrounding collagen [58]. Contraction occurs due to iterative cycles of pseudopodia extension, focal adhesion development, collagen adhesion, pseudopodia retraction and collagen release via a mechanism dependent on the integrins, actin stress fibre development and myosin II assembly [57], [59].

Contraction in response to bradykinin was attenuated when cells were treated with a broad spectrum MMP inhibitor and siRNAs against MMP-1, suggesting MMP-1 contributes to contraction in a non-redundant manner. MMP-1 localises to the tips of pseudopodia and collagen proteolysis has been shown to promote the formation of pseudopodia, collagen reorganisation and focal adhesion destruction [60], [61]. This suggests that MMP-1 promotes the dynamic behaviour required to elicit gel contraction. Consistent with these findings, *in vitro* studies of ASM collagen gels suggest that MMP-1 can alter the structure of collagen fibrils [17] and that asthma derived ASM is more contractile in this system [23], [36]. Furthermore, acetylcholine induced airway contraction and contraction velocity within murine lung slices was enhanced by collagenase incubation [19], which was attributed to a decrease in airway wall stiffness and collagen degradation. As in our study, the effect of collagenase in intact airways had a finite but physiologically relevant effect on contraction which suggests that the effect is one of a number of factors contributing to airway narrowing.

Taken together, these findings suggest that, the induction of MMP-1 by abnormal ECM in the airways of patients with asthma may result in reduced airway stiffness, enhanced airway narrowing in response to bronchoconstrictor stimuli and worsening asthma symptoms. The absence of tenascin-C in control tissue, deposition in asthma and proximity to MMP-1 expression denote tenascin-C as a potential, pathologically-relevant, regulator of MMP-1 expression *in vivo* and target for therapeutic intervention against airway remodelling. Further studies in human asthma are required to determine whether this mechanism contributes to bronchial hyper-responsiveness and if so, how the expression and activation of MMP-1 is driven by pro-inflammatory factors during exacerbations and the effect of asthma treatment, particularly corticosteroids.
